# The Influence of Solvents and Colloidal Particles on the Efficiency of Molecular Antioxidants

**DOI:** 10.3390/antiox12010099

**Published:** 2022-12-31

**Authors:** Bojana Katana, Kata Panna Kókai, Szilárd Sáringer, Adél Szerlauth, Dóra Takács, István Szilágyi

**Affiliations:** MTA-SZTE Lendület Biocolloids Research Group, Interdisciplinary Excellence Center, Department of Physical Chemistry and Materials Science, University of Szeged, H-6720 Szeged, Hungary

**Keywords:** molecular antioxidants, solubility, latex, colloidal dispersion, DPPH

## Abstract

The radical scavenging activity of three molecular antioxidants (trolox, rutin and ellagic acid) was investigated in different solvents with and without added polymer-based colloidal particles (SL-IP-2). Rutin and ellagic acid showed poor solubility in water, preventing the accurate measurement of the effective antioxidant concentration values, which were determined in ethanol/water (EtOH/H_2_O) mixtures. The presence of trolox and rutin changed neither the surface charge properties nor the size of SL-IP-2 in these solvents, while significant adsorption on SL-IP-2 was observed for ellagic acid leading to overcharging and rapid particle aggregation at appropriately high antioxidant concentrations in EtOH/H_2_O. The differences in the radical scavenging capacity of trolox and ellagic acid that was observed in homogeneous solutions using water or EtOH/H_2_O as solvents vanished in the presence of the particles. Rutin lost its activity after addition of SL-IP-2 due to the larger molecular size and lower exposure of the functional groups to the substrate upon interaction with the particles. The obtained results shed light on the importance of the type of solvent and particle–antioxidant interfacial effects on the radical decomposition ability of molecular antioxidants, which is of crucial importance in industrial processes involving heterogeneous systems.

## 1. Introduction

Oxidative stress is a condition caused by the disproportion between the production of reactive oxygen (ROS) and nitrogen (RNS) species and the organism’s ability to neutralize these reactive substances [1,2]. ROS and RNS react with cellular compounds such as lipids and proteins leading to the disruption of vital functions and many other pathological conditions [3]. Oxidative stress-induced damage has already been demonstrated during the development of several chronic health problems, such as neurodegenerative (e.g., Parkinson’s, Alzheimer’s, and Huntington’s diseases), cardiovascular and inflammatory diseases, as well as cancer [4,5,6]. Apart from their harmful effects, these reactive substances are involved in essential biological processes, e.g., in the removal of various infectious agents and in cell signaling. Therefore, the concentration of ROS and RNS must be optimized, and nature has assigned this role to antioxidants [7].

Antioxidants are compounds that can delay or inhibit the damage of any substance from the reactive species mentioned above [8,9,10,11,12]. While some antioxidants directly neutralize ROS or RNS derivatives, others prevent their production by binding to metal ions or by inhibiting the action of relevant enzymes [13]. Both molecular (exogenous) and enzymatic (endogenous) antioxidants exist to combat oxidative stress [14]. The latter ones, including superoxide dismutase, catalase, and various peroxidases, are the most effective defense systems that act in cascade reactions in the cellular environment [12]. Due to the high sensitivity of enzymatic antioxidants to changes in pH, temperature, and pressure, molecular antioxidants are increasingly being used in antioxidant treatments in both medical and industrial processes [11,15]. These include flavonoids, polyphenols, and vitamins that can be ingested. For instance, vitamins, especially ascorbic acid (vitamin C), are broad-spectrum antioxidants involved in the neutralization of hydroxyl, alkoxy, and superoxide radicals [16]. In addition, tocopherol (vitamin E) prevents lipid peroxidation in the cell membranes by forming a compound of less reactivity with ROS and RNS. An analogue of vitamin E is trolox, which also counteracts the damaging effects of oxidative stress [8]. Moreover, it is often used as a standard for measuring the antioxidant capacity of foods and beverages [17]. Polyphenols are secondary metabolites of plants having unique physical and chemical properties, such as the ability to complex with certain proteins, polysaccharides, and metal ions. In addition, their antioxidant, anti-inflammatory, antiviral, and antimutagenic effects are known [18]. Ellagic acid also belongs to the group of polyphenols within antioxidants, and it is present in tropical and berry fruits, or in nuts, while serving as an anti-inflammatory, antidiabetic, and antiviral agent [19,20,21]. The group of polyphenols contains flavonoids as well, such as rutin, which prevents the binding of hydrogen peroxide to iron(II) ions in the blood stream and thus, hinders the formation of cell-damaging free radicals [22]. Moreover, it reduces the risk of the development of heart diseases and strengthens capillaries, thereby, reducing bleeding [23]. Advances in science and technology allow the production of certain molecular antioxidants at an industrial scale nowadays [24].

Major obstacles to the application of molecular antioxidants include poor water solubility or structural sensitivity, which can be eliminated by embedding them into composite materials [25]. Recently, many studies dealt with the immobilization of exogenous antioxidants on solid supports. Accordingly, various layered, tubular, or porous materials have been used and several methods (e.g., physical adsorption, intercalation, and covalent grafting) are known for the attachment of the antioxidant molecules to surfaces. Carnosine and gallic acid were intercalated between layers of layered double hydroxides (LDHs) by ion exchange and coprecipitation and their antioxidant activity was tested by the efficiency in the reduction of 2,2-diphenyl-1-picrylhydrazyl (DPPH), a widely used model radical [26,27]. Glutathione was fixed between the layers of montmorillonite [28], while curcumin was covalently linked to the outer surface of halloysite nanotubes [29]. It has been demonstrated that trolox immobilized between the LDH lamellae retained its antioxidant activity [30], while entrapment inside the porous structure of silica conserved its radical scavenging capacity, and the silica matrix stabilized and preserved the molecular structure of trolox [31]. An LDH-based hybrid was prepared with polyphenolic ellagic acid as radical scavenger. By using the coprecipitation process, ellagic acid was specifically intercalated into LDHs, and the resulting hybrids possessed strong antioxidant activity confirmed by their ability to scavenge DPPH radicals [25]. LDHs were also used as carriers for folic acid and in vitro antioxidant activity assessments revealed that the developed folic acid-LDH composite system decomposed DPPH and hydroxyl radicals with remarkable scavenging activity [32]. Additionally, it was reported that resveratrol immobilized in monodisperse cyano-functionalized porous particles retained its bioactivity, and the measured antioxidant activity was higher compared to pure resveratrol [33]. Here, the cyano-functional groups were incorporated into the porous particles and effectively stabilized the structure of the antioxidant.

The industrial application of immobilized molecular antioxidants has become widespread [9,15,24]. For example, modifying packaging materials with antioxidant agents is an effective way to increase durability. The antioxidant and fungicidal activities of composites consisting of poly(3-hydroxybutyrate), starch, and eugenol have been investigated and have been found to be excellent additives in packaging materials, giving rise to the increased shelf-life of food [10]. In addition, curcumin-modified zein proteins possessed long-term stability, biocompatibility, and significant antioxidant efficacy, which make the composite useful in various food processes [34]. Composite nanoparticles containing hydrophilic additives were prepared using the supercritical antisolvent process and they were used as carriers for resveratrol loading, which resulted in an increase in the solubility of resveratrol. The developed nanocomposites proved to be promising formulation additives in healthcare and other cosmetic products [35]. Ellagic acid was intercalated into LDHs and like the free ellagic acid, the obtained nanohybrid demonstrated a modest effect on the hepatocyte cells, as revealed in in vitro bioassays, which indicated a possible application in testing cancer cell lines [36]. Rutin was encapsulated inside mesoporous silica materials, where the drug loading was performed by the solvent impregnation method [37]. Such an immobilization process allowed long-term physical stability as well as improved kinetic saturation solubility and thus, the resulting system can be considered as a promising oral delivery agent.

The activity of immobilized antioxidants depends strongly on the surface area available in the dispersions, which can be improved by colloidal stabilization of the composite particles with synthetic [38,39] or natural [40,41,42] polymers, which are able to strongly adsorb on oppositely charged surfaces [43]. For instance, the colloidal control and surface functionalization of an LDH-antioxidant nanocomposite were studied [44]. Namely, three different cationic polymers such as polyethyleneimine, protamine sulfate, and poly(acrylamide-co-diallyl dimethylammonium chloride) were used to functionalize the LDH surface and the adsorbed layers enhanced the colloidal stability of the composites changing also the radical decomposing activity of the obtained hybrids. The developed nanocomposites revealed long-term radical capturing capacity and can be considered as highly active antioxidant colloids. Nevertheless, apart from some pioneering studies, no comprehensive data are available on the relationship between the colloidal stability and antioxidant capacity of immobilized molecular radical scavengers.

**Scheme 1:**
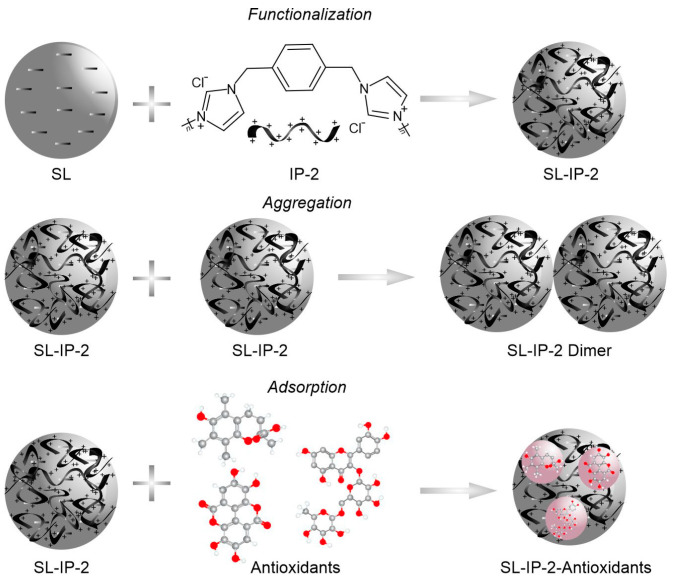
Illustration of the surface functionalization of SL with IP-2 (**top**), particle aggregation (**middle**) and possible antioxidant adsorption (**bottom**) processes.

The aim of the present work was to investigate the activities of selected molecular antioxidants (trolox, rutin, and ellagic acid) in different solvents and in the presence of polymer-modified lattices, which were designed and prepared by systematic optimization of the colloidal stability of the particles (Scheme 1). Trolox is a standard exogeneous antioxidant, while applying rutin and ellagic acid allowed us to study the effect of molecular mass on the potential adsorption processes on the particles. Potential changes in surface charge and aggregation tendency were explored by light scattering techniques in both water and EtOH/H_2_O media. To assess the activity of antioxidants, the DPPH test was performed in the presence and absence of the particles in different solvents. Overall, the main goal was to investigate the solvent-antioxidant-particle interactions at the interface, which have an important role in the solubilization and structural stabilization of molecular antioxidants in heterogeneous systems.

## 2. Materials and Methods

Styrene (99%, extra pure, stabilized), antioxidants (trolox (97%), rutin (97+%), ellagic acid (≥97%)), and polyvinylpyrrolidone (PVP, K-30, Mw 50,000) were purchased from Acros Organics (Geel, Belgium). The 2,2-diphenyl-1-picrylhydrazyl (DPPH) radical (95%) was bought from Alfa Aesar (Kandel, Germany). Potassium chloride (KCl), potassium peroxodisulphate (KPS), ethanol, and methanol were acquired from VWR (Debrecen, Hungary). Nitrogen (4.5) gas was obtained from Messer (Budapest, Hungary). All chemicals were used without further purification. Ultrapure water with a resistivity higher than 18.2 MΩ∙cm was obtained from a three-stage VWR Puranity TU+ (Debrecen, Hungary) device. The measurements were carried out at 25 °C and at pH 10 unless otherwise indicated.

### 2.1. Synthesis of Polystyrene Spheres

Anionic sulfate functionalized latex spheres (SL) were prepared by emulsifier-free emulsion polymerization using KPS as a charge initiator. This method is based on a previously reported protocol with a slight modification [45]. Briefly, 12.1 g of styrene monomer and 0.18 g of PVP were added to 100 mL of deionized water in a 250 mL three-neck round bottom flask placed in an oil bath under nitrogen atmosphere at room temperature. The mixture was stirred at 400 rpm for 30 min and the temperature was gradually increased to 70 °C. Thereafter, 0.3 g of KPS dissolved in 20 mL of deionized water was added to the reaction mixture and the temperature was kept at 70 °C for 24 h. The dispersion was finally allowed to cool down to room temperature. The residual styrene and PVP were removed by repeated washing/centrifugation/redispersing steps using 2-time water, 2-time ethanol, and again 2-time water as solvent. The obtained latex spheres were dialyzed against water for 2 days. The concentration of the final stock was 80 g/L, which was diluted in the experiments.

The detailed synthesis of the polyimidazolium-based polymer (IP-2) has been reported earlier [46]. The IP-2 functionalized SL stock dispersion was prepared by simple mixing calculated volumes of 1 g/L IP-2 solution and an SL particle stock dispersion of 10 g/L concentration.

### 2.2. Dynamic Light Scattering

Dynamic light scattering (DLS) was used to measure the hydrodynamic radius (*R_h_*) of the particles. The experiments were carried out with a Litesizer 500 instrument (Anton Paar) at a scattering angle of 175°. This device is equipped with a 40 mW semiconductor laser operating at 658 nm wavelength. The correlation function was fitted with the Cumulant method to obtain the decay rate constant (*Γ*), and the diffusion coefficient (*D*) was calculated as follows [47]:

(1) $$D=\Gamma /q^{2}$$

where *q* is the scattering vector, which can be calculated using the parameters of the experimental setup as:

(2) q=(4πn/λ)sin(Θ/2)

where *n* is the refractive index of the medium, *λ* is the wavelength of the laser beam and Θ is the scattering angle. The *R_h_* was then calculated by the Stokes-Einstein equation [48]:

(3) $$R_{h}=k_{B}T/6\eta \pi D$$

where *k_B_* is the Boltzmann constant, *η* is the dynamic viscosity, and *T* is the absolute temperature. The samples were prepared in disposable cuvettes (VWR) by mixing the appropriate amount of the IP-2 stock, antioxidant solution, water, or EtOH/H_2_O and then, SL-IP-2 particles were added in calculated amount from the stock dispersion to reach the final concentration of 10 mg/L. The samples were allowed to rest for 2 h and then equilibrated for 30 s in the instrument before data collection was started. Time-resolved DLS measurements were performed to determine the stability ratio (*W*) values in the SL-IP-2 systems as follows [49]:

(4) $$W=\frac{k_{fast}}{k}$$

where *k* is the apparent aggregation rate constant calculated from the increase in the *R_h_* over time and *k_fast_* is the rate constant determined in 1 M KCl solutions, in which fast aggregation of the particles occurs since the electrostatic interparticle repulsion is screened under this experimental condition. Thus, stability ratios close to unity indicate unstable samples and higher values refer to more stable dispersions and slower particle aggregation [50].

### 2.3. Electrophoresis

The electrophoretic light scattering measurements were performed with the same instrument as the one used for the DLS. The preparation of the dispersions was identical to the protocol applied in the DLS experiments. The samples were allowed to rest overnight before recording the electrophoretic mobility data, which was carried out in 700 µL omega-shaped plastic cuvettes (Anton Paar).

### 2.4. Determination of Antioxidant Concentration

Trolox, rutin and ellagic acid absorption spectra were recorded in the range of 200–600 nm wavelength in 10 mm polystyrene cuvettes (VWR) with a Genesys 10S spectrophotometer (Waltham, MA, USA). The total volume of the measured antioxidant solutions was 3 mL using water or EtOH/H_2_O (1:1) mixtures as solvent. Calibration curves were recorded by reading the absorbance values at wavelengths, at which the absorption spectra showed maxima. The relationship between the concentration (*c*) of the antioxidant and the absorbance ($A_{\lambda }$) measured at a given wavelength is described by the Lambert–Beer law:

(5) $$A_{\lambda }=\varepsilon_{\lambda }cl$$

where $\varepsilon_{\lambda }$ is the molar absorbance coefficient and l is the optical pathlength. Note that this law applies only to diluted solutions, where intermolecular interactions are negligible.

#### Scavenge of DPPH Radicals

The antioxidant activity was probed by a standard assay based on the reduction of DPPH radicals and the corresponding decrease in the intensity of purple color [51]. Accordingly, 3500 µL of 24 mg/L (60 µM) methanolic DPPH solution was mixed with antioxidant solutions of different volumes (10–100 µL) followed by the addition of methanol to reach the total volume of 3600 µL. Antioxidant solutions and antioxidant-SL-IP-2 stock dispersions were prepared in water and EtOH/H_2_O media. During the reaction, the transformation from the oxidized (purple) to the reduced (yellow) form of the DPPH was followed by measuring the absorbance at 517 nm wavelength with a VWR V-1200 spectrophotometer using disposable polystyrene cuvettes (VWR). Ultrapure water (100 µL) was added instead of antioxidants in the control experiment, however, change in the absorbance was negligible in this case. To determine the concentration of radicals that remained in the solution at the end of the reaction (DPPH%), the absorbance values measured at the final state (A) were divided by the initial absorbance (A_0_) as:

(6) $$\mathrm{DPPH}\%=\frac{A}{A_{0}}100\%$$

The obtained DPPH% values in percentage were used later to determine the effective concentration (EC_50_), which corresponds to the antioxidant concentration needed to decompose 50% of the DPPH radicals present in the test solution. Accordingly, EC_50_ values were calculated by plotting the DPPH% values versus the antioxidant concentrations. The average error of these measurements is about 5%. The absorbance values for the samples containing SL-IP-2 were corrected with the one derived from the particle-induced light scattering.

## 3. Results and Discussion

### 3.1. Development of SL-IP-2 Particles

**Figure 1:**
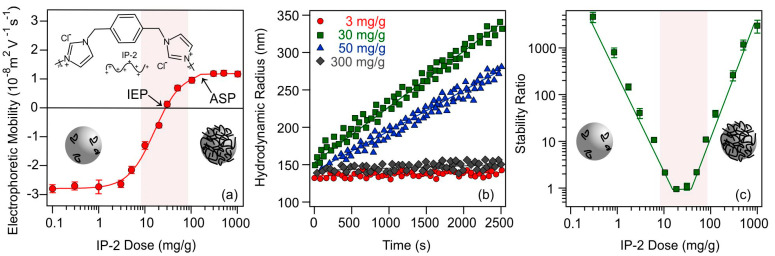
Electrophoretic mobility of SL at different IP-2 polymer doses: (**a**); hydrodynamic radius versus time (**b**); and stability ratios of SL particles as a function of the IP-2 dose (**c**). The measurements were performed at pH 10 and at 10 mM ionic strength adjusted by KCl, while 10 mg/L SL concentration was applied. The mg/g unit refers to mg IP-2 per one gram of SL. The solid lines in (**a**,**c**) are eye guides, while in (**b**), they are linear fits to calculate apparent rate constants and stability ratios (Equation (4)) shown in (**c**).

To prepare the SL-IP-2 composite dispersions, the imidazolium-based polymer (see structure in Figure 1a) was adsorbed on SL particles, which are negatively charged under the experimental conditions applied due to the ionized sulfate group, whereas the IP-2 polymer bears a permanent positive charge. The main goal of this study was to determine the SL-to-IP-2 ratio, under which their composite forms stable dispersions and possess positive charges to facilitate the adsorption of the molecular antioxidants [44]. To assess surface charge features, the electrophoretic mobilities of SL were measured at different IP-2 doses (Figure 1a).

At low IP-2 doses, negative electrophoretic mobility values were measured due to the negatively charged surface of the SL particles. As the concentration of IP-2 increased, the mobilities gradually increased until they reached zero, where charge neutralization occurred due to the adsorption of the IP-2 on the oppositely charged SL surface. The so-called isoelectric point (IEP) was measured at a dose of 30 mg/g IP-2. Further increase in the polymer loading resulted in the development of positive particle charge beyond the IEP. Such a charge inversion usually occurs in oppositely charged particle–polymer systems due to electrostatic attraction, the increase in entropy accompanying the adsorption of IP-2 and subsequent release of solvent molecules and counterions, and ion correlation forces [43,52,53]. Further addition of the IP-2 no longer results in a change in the electrophoretic mobility values and the adsorption saturation plateau (ASP) was reached. Putting more polymer above the ASP does not cause adsorption due to the saturated surface and the excess polymer remains in the solution.

The effect of IP-2 concentration variation on the aggregation tendency of SL particles is shown in Figure 1b. At 3 mg/g dose, where the particles are negatively charged (Figure 1a), the *R_h_* changes only slightly over time, the particle aggregation is slow, and an intermediate stability ratio can be calculated (Figure 1c). As the polymer dose increases to 30 mg/g, the slope of the *R_h_* versus time plot increases dramatically leading to a stability ratio close to unity. Such a rapid aggregation is observed at the IEP. As the dose is further increased to 50 mg/g, where the particles possess positive charge due to overcharging, moderately fast aggregation occurs, while stable dispersions with very slow particle aggregation were experienced at 300 mg/g IP-2 loading, above the ASP.

Comparing the charge and aggregation tendencies by altering the SL-to-IP-2 ratio in the samples, it can be stated that the dispersions are unstable near the IEP, while sufficiently charged particles below and above the IEP are more stable. These results imply that the IP-2 dose-dependent colloidal stability of SL can be interpreted with the classical DLVO (Derjaguin, Landau, Verwey and Overbeek) theory [54,55,56,57], which predicts that the overall interparticle forces are determined by the sum of repulsive electrical double layer and attractive van der Waals forces. Indeed, rapid aggregation is observed in the present system near the IEP, under which conditions the van der Waals attractive forces predominate over the weak or even vanished repulsive forces otherwise originating from the overlapping electrical double layers. However, at lower and higher doses, where the surface charge of the particle is high, dispersions are stable due to strong electrostatic repulsion between the double layers. In addition, the steric stabilizing effect of the adsorbed polymer chains [58,59,60] cannot be ruled out, while further studies are needed to unambiguously confirm their presence.

Based on the above findings, a dose of 300 mg IP-2 per 1 g SL particle was used to functionalize the SL particles for further studies. At this concentration, it is possible to prepare stable dispersions of the SL-IP-2 particles, which possess a significantly high positive charge to possibly facilitate the adsorption of molecular antioxidants discussed later.

### 3.2. Solubility of Antioxidants

The antioxidant solutions were serially diluted and the absorbances were recorded in the 200–600 nm range. Based on the spectra, the wavelengths corresponding to the absorption maximum were read for each antioxidant. This wavelength was found to be 290 nm for trolox, 378 nm for rutin, and 359 nm for ellagic acid. At these wavelengths, the absorbance values for the different concentrations were read and plotted (Figure S1 in the Supplementary Materials). Linear fits were performed on the points to establish the calibration curves. In aqueous solutions, linearities were observed until 100 mg/L for trolox, 75 mg/L for rutin, and 10 mg/L for ellagic acid. In the EtOH/H_2_O medium, the linear range was observed up to 100 mg/L for trolox and rutin, and 75 mg/L for ellagic acid. These data indicate the better solubility of rutin and ellagic acid in EtOH/H_2_O. The almost identical absorbance values for trolox justify the suitability of this antioxidant as a standard in DPPH tests [17,61].

### 3.3. Radical Scavenging Activity of Antioxidants

**Figure 2:**
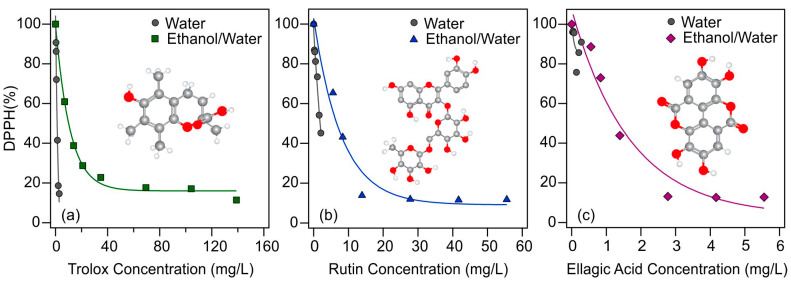
DPPH scavenging activity of antioxidants (trolox (**a**); rutin (**b**); and ellagic acid (**c**)) as a function of their actual concentration in the assays using different solvents (water (gray circles for all) and ethanol/water (green squares, blue triangles, and purple diamonds, respectively)) in the stock solutions of the antioxidants. The solid lines are mathematical functions used to calculate the EC_50_ values.

The tendencies in the antioxidant activities upon changing the experimental conditions (i.e., solvent composition or addition of particles) were investigated by assessing the DPPH radical scavenging activities of the systems. In the classical DPPH tests [51], the radicals are reduced by the antioxidants in the solution, which turns the initial purple color (DPPH radical) of the solution to yellow (reaction product). This is the basic phenomenon allowing to monitor the reaction spectrophotometrically at 517 nm wavelength. The reaction between the radical and the antioxidant is initially rapid, and after a while, the absorbance returns to a constant value once the reaction is complete. Using the absorbance data measured after 45 min, Equation (6) was used to calculate the percentage of radicals that remained in the solution, which were plotted as a function of the initial antioxidant concentration (Figure 2).

The measured DPPH% data indicate progressive radical scavenging with increasing antioxidant concentrations in all cases. While the effective concentrations in the aqueous solutions could not be reliably determined for all three molecular antioxidants, likely due to the limited solubility, the EC_50_ values were obtained in the EtOH/H_2_O samples. For rutin, nearly 90% of the radicals were decomposed in this solvent, while the maximum applicable antioxidant concentration in the aqueous solution neutralized about 60% of the radicals. A similar phenomenon can be observed for ellagic acid, about 10% of the radicals remained in solution after an appropriately high concentration was used in EtOH/H_2_O solution, while 80% of the radicals did not react in aqueous solution. Trolox behaved differently, as even its low concentration was enough to neutralize nearly 80% of the DPPH in both cases. These findings shed light on the importance of the type of solvent and indicate that increased antioxidant activity for rutin and ellagic acid could be attained in ethanolic solutions, whereas this is not possible in water due to the limited solubility.

### 3.4. Interaction between Antioxidants and SL-IP-2

Characteristic size and charging data for SL-IP-2 as well as for the bare SL particles are presented in Table S1 in the Supplementary Materials. It can be concluded that by applying an IP-2 dose of 300 mg/g, the formed SL-IP-2 particles possess slightly higher hydrodynamic size compared to bare SL, most likely due to the adsorbed polymer layer, which contributes to the *R_h_* with a few nm in line with literature data reported earlier with oppositely charged particle-polymer systems [43]. The slightly higher polydispersity index (PDI) values for SL-IP-2 originate from the fact that the system goes through the IEP in a transient-like fashion and particle aggregation occurs for a short period, while the dispersion is stable before and after the IP-2 addition. Even this time is long enough for the formation of some aggregates giving rise to a higher PDI value [62].

**Figure 3:**
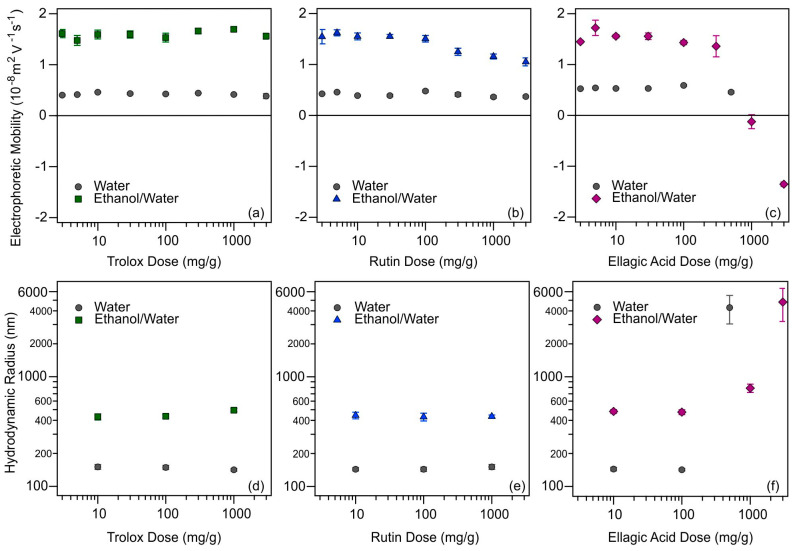
Electrophoretic mobilities of SL-IP-2 particles as a function of the dose of trolox (**a**); rutin (**b**); and ellagic acid (**c**). The *R_h_* of SL-IP-2 versus trolox (**d**); rutin (**e**); and ellagic acid (**f**) concentrations. Data determined in different solvents, water (gray circles) and ethanol/water (green squares, blue triangles, and purple diamonds, respectively) are presented. The concentration of the SL-IP-2 particles was kept at 10 mg/L.

The possible adsorption of antioxidants on the SL-IP-2 particles was investigated by electrophoretic mobility measurements. The amount of SL-IP-2 was kept constant, while the concentration of antioxidants was varied and its effect on the charge of the particles was examined. The electrophoretic mobilities of SL-IP-2 as a function of the antioxidant concentration are shown in Figure 3a–c.

Based on the mobilities measured at low antioxidant concentrations, one can conclude that adsorption was more likely to occur in aqueous media under these experimental conditions. This is indicated by the smaller electrophoretic mobility values determined in the water below 100 mg/g antioxidant dose, which also indicates that the antioxidant is adsorbed in a partially or fully deprotonated (ionized) form. Another confirmation of the adsorption is that these mobilities are lower than the one obtained for the SL-IP-2 without added antioxidants (Table S1 in the Supplementary Materials). The mobility measured for SL-IP-2 in EtOH/H_2_O without added antioxidants (1.84 × 10^−8^ m^2^/Vs) was about the same as the one obtained for SL-IP-2 in water and very similar values were determined in the presence of the antioxidants at low concentrations. For trolox (Figure 3a), no change in the mobility data was detected by increasing the concentration in either solvent due to its good solubility. Very similar tendencies were observed for rutin (Figure 3b), however, the slight decrease in mobilities at high concentrations indicates that the adsorption process took place to some extent in EtOH/H_2_O mixture. The behavior of ellagic acid samples was very similar to trolox and rutin until 300 mg/g (Figure 3c) and the same conclusions could be made. Nevertheless, in the case of ellagic acid in EtOH/H_2_O, the electrophoretic mobility of the particles decreased significantly above this concentration, they reached IEP around 1000 mg/g, while overcharging was observed at high concentrations. These results imply that ellagic acid adsorbed on the surface of the particle in a remarkable amount in EtOH/H_2_O, in contrast to the water samples. Given the poorer solubility in the latter solvent (Figure S1e in the Supplementary Materials), this finding is rather surprising, since one may expect that ellagic acid does not prefer adsorption from ethanolic solution due to its good solvation. Similar overcharging phenomenon was reported earlier, where ellagic acid adsorption on positively charged clay particles took place in significant quantities [44].

The *R_h_* values of the particles determined at antioxidant doses of 10, 100, and 1000 mg/g are shown in Figure 3d–f (some intensity correlation functions and cumulant fits are presented in Figure S2 in the Supplementary Materials). For trolox (Figure 3d) and rutin (Figure 3e), the *R_h_* changed neither in water nor in EtOH/H_2_O with increasing the concentration, but a significant difference was observed between the hydrodynamic sizes obtained in aqueous and EtOH/H_2_O solutions. While values like the original SL-IP-2 particle size (Table S1 in the Supplementary Materials) were obtained in the aqueous dispersions, larger *R_h_* data were measured in the EtOH/H_2_O medium in the presence of these antioxidants. This might be explained by the fact that the Debye–Hückel parameter clearly depends on the solution composition and the decrease in the permittivity with an increasing EtOH molar fraction causes a reduction in the degree of dissociation of the surface functional groups [63,64]. Such a reduced relative permittivity by the EtOH addition leads to weaker repulsion by the electrical double layers [65] and consequently, to the formation of some particle aggregates with higher hydrodynamic sizes. For the systems containing ellagic acid (Figure 3f), the above findings were obtained until 300 mg/g concentration. However, the *R_h_* changed greatly due to the significant aggregation of the particles at higher doses. Comparing the tendencies in the mobility and size data, charge neutralization and overcharging occurred in this concentration regime owing to the significant adsorption of ionized ellagic acid molecules on the SL-IP-2 in EtOH/H_2_O. Indeed, DLVO theory predicts rapid particle aggregation around the IEP due to vanishing electrical double layer forces [43,53] and such aggregation is responsible for the high *R_h_*.

Based on the above results, an antioxidant dose of 100 mg per 1 g of SL-IP-2 was selected for the DPPH scavenging activity measurements carried out in dispersions at different particle concentrations, but at the above-mentioned relative amount of the antioxidants. Table S2 in the Supplementary Materials shows the characteristic *R_h_*, PDI, and electrophoretic mobility of SL-IP-2 measured under this experimental condition, together with performance indicators in the DPPH decomposition tests.

### 3.5. Antioxidant Activity of the Colloidal Systems

**Figure 4:**
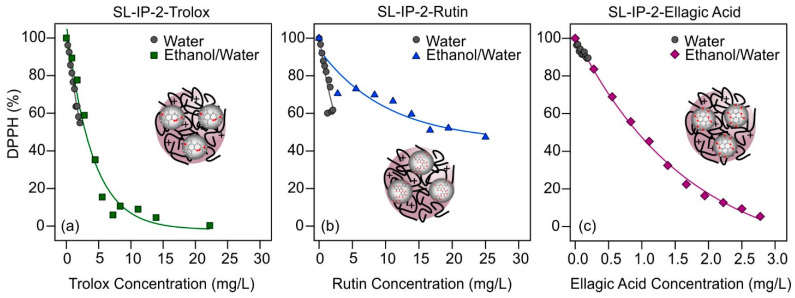
DPPH scavenging activity of SL-IP-2-trolox (**a**); SL-IP-2-rutin (**b**); and SL-IP-2-ellagic acid (**c**) as a function of the actual antioxidant concentrations using different solvents (water (gray circles) and ethanol/water (green squares, blue triangles, and purple diamonds, respectively,)) for stock dispersion preparation. The relative amount of the antioxidants was always 100 mg per 1 g of SL-IP-2. The solid lines are mathematical functions used to calculate the EC_50_ values. The schemes in inset illustrate the possible antioxidant adsorption on the surface of the polymer coated particles.

Regarding the radical scavenging activity of antioxidants when adsorbed on the surface of SL-IP-2 at a concentration of 100 mg/g (Figure 4), similar overall tendencies were observed as in the case of pure antioxidant solutions (Figure 2).

**Figure 5:**
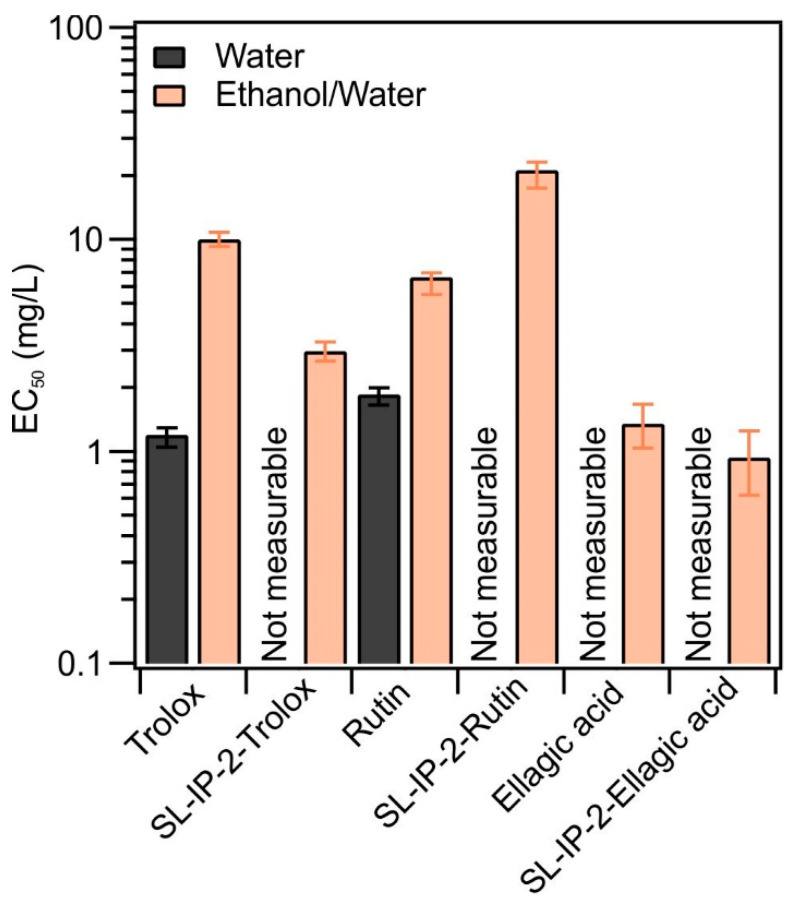
Effective concentrations measured in homogeneous solutions of the antioxidants and in the presence of SL-IP-2 in different solvents (water (dark bars) and ethanol/water (light bars)).

Accordingly, in water media, antioxidants neutralized about 10–40% of the radicals at the highest applicable concentration, while in EtOH/H_2_O, above appropriately elevated loadings, DPPH remains in solution only in small amounts. However, rutin behaved somewhat differently (Figure 4b) and showed a smaller antioxidant effect in the presence of the particles than the native antioxidant form in homogeneous solution, giving rise to a larger DPPH% value at high concentrations (Table S2 in the Supplementary Materials). It is assumed that the rutin is immobilized on the surface of the particle in such a way that its reactive groups are less accessible to the radicals and thus, lower DPPH scavenging activity can be detected. The striking difference observed in the data obtained for trolox and ellagic acid is that the system-specific DPPH% values are in good agreement in either solvent at low antioxidant concentrations in the presence of SL-IP-2 (Figure 4a,c), while they are remarkably different in the absence of the particles (Figure 2). This result also indicates that interfacial interactions most likely took place even if the size or charging data were not sensitive to them. The effective concentration values determined in the different systems (with and without particles) are shown in Figure 5.

The EC_50_ data indicate that, with two exceptions, no EC_50_ could be determined in aqueous media due to the limited solubility of the antioxidants. However, in the EtOH/H_2_O mixture, the EC_50_ values decreased for trolox and ellagic acid, indicating higher antioxidant efficiencies in the presence of SL-IP-2. This finding clearly implies that the interfacial interaction took place in these systems, which improved the radical scavenging ability of the molecular antioxidants. This phenomenon can be explained by the fact that the antioxidants were fixed to the surface of the particles in such a way that the functional groups responsible for the antioxidant effect became more readily available to the DPPH radical. In addition, the carrier particles may attract the substrate and hence, bring the radicals to the vicinity of the surface, at which the antioxidants are present in higher concentrations. Similar results were reported earlier for ellagic acid in the presence of clay particles, where surface adsorption did not compromise the antioxidant activity [25]. Nevertheless, for rutin, due to the significant size difference compared to trolox and ellagic acid, not all functional groups of the larger rutin molecules are available after interaction with the SL-IP-2 surface, and this steric hindrance caused lower antioxidant activity reflected in higher DPPH% and EC_50_ values.

## 4. Conclusions

The activities of three molecular antioxidants, trolox, rutin, and ellagic acid, were determined in aqueous and EtOH/H_2_O media, in the absence and presence of colloidal particles (SL-IP-2) to investigate the effect of their potential interfacial behavior on the antioxidant activity. The solubility of the antioxidants was assessed in both solvents, and the results indicate a better solubility of rutin and ellagic acid in the EtOH/H_2_O medium, while almost identical absorbance values were obtained for trolox in both media.

The colloidal particles consisted of SL functionalized with oppositely charged imidazolium-based IP-2 polymer. The experimental conditions were optimized to form stable dispersions of positively charged SL-IP-2 particles. Electrophoretic mobility data determined at different antioxidant concentrations revealed that the adsorption of the molecules likely occurred on the SL-IP-2 surface, while its extent was system specific. Such adsorption caused significant changes in the charge of the particles in the aqueous medium, while the antioxidant concentration did not affect the surface charge in the EtOH/H_2_O mixture. Ellagic acid behaved differently, and its adsorption led to charge neutralization and overcharging of the particles in ethanolic solutions and to particle aggregation at higher concentrations.

The DPPH scavenging ability of the antioxidants was examined in homogeneous solutions and in dispersions containing SL-IP-2. While effective concentrations in purely aqueous media could be determined in only two cases, they were measured in EtOH/H_2_O for each system due to the different solubilities. Ellagic acid and trolox showed higher antioxidant activity in the presence of the colloidal particles due to advantageous interfacial interactions giving rise to more available functional groups, which, combined with the attraction of the substrate by SL-IP-2, led to lower EC_50_ values. For rutin, this trend was reversed, due to its larger size and sterically hindered functional groups.

Overall, it can be concluded that the medium used had a great influence on the solubility and subsequent activity of the antioxidants. Another important conclusion is that the radical scavenging efficiency can be affected by the addition of appropriate colloidal particles. Such an effect depends on the charge, size, and surface functionalities of the particles as well as on the structure of the antioxidant molecules. The obtained results should attract considerable interest, especially in industrial applications (e.g., manufacturing of foods, cosmetics, and plastics), where efficient antioxidant agents are required for better quality products, while colloidal particles are also present in the systems. The appropriate combination of molecular antioxidants and particles may result in more effective radical scavenging activity.

## Data Availability

Data is contained within the article and Supplementary Material.

## Supplementary Materials

The following supporting information can be downloaded at: https://www.mdpi.com/article/10.3390/antiox12010099/s1, Figure S1: Calibration curves for antioxidants in water (left column) and in EtOH/water (right column) obtained at the wavelength corresponding to the absorption maximum recorded for each antioxidant.; Figure S2: Intensity correlation function (g^2^) versus the delay time for SL-IP-2-Trolox, SL-IP-2-Rutin, and SL-IP-2-Ellagic acid systems in water (a–c), and in ethanol/water (d–f). The antioxidant dose in all systems was maintained at 100 mg/g. The red solid line refers to the correlation function, and blue solid line represents the Cumulant fit. Table S1: Characteristic size and charge data of the bare SL particles and SL-IP-2.; Table S2: The hydrodynamic radius (*R_h_*), polydispersity index (PDI), and electrophoretic mobility (EM) data of SL-IP-2 with 100 mg/g dose of antioxidants. Effective concentrations and minimum DPPH% values are also presented.
