# Supplementary material for: The Influence of Solvents and Colloidal Particles on the Efficiency of Molecular Antioxidants
Source: Antioxidants (Basel). 2022 Dec 31;12(1):99. doi: 10.3390/antiox12010099 (PMC9855148; doi:10.3390/antiox12010099)
Supplement: Supplementary file 1 [file antioxidants-12-00099-s001.zip › antioxidants-2087497-supplementary.pdf]

Supplementary Materials (SM)

The Influence of Solvents and Colloidal Particles on the Efficiency of Molecular Antioxidants

Bojana Katana[1], Kata Panna Kókai[1], Szilárd Sáringer[1], Adél Szerlauth[1], Dóra Takács[1], and István Szilágyi[1,*]

[1]MTA-SZTE Lendület Biocolloids Research Group, Department of Physical Chemistry and Materials Science, Interdisciplinary Excellence Center, University of Szeged, H-6720 Szeged, Hungary

*Corresponding author. Email: szistvan@chem.u-szeged.hu

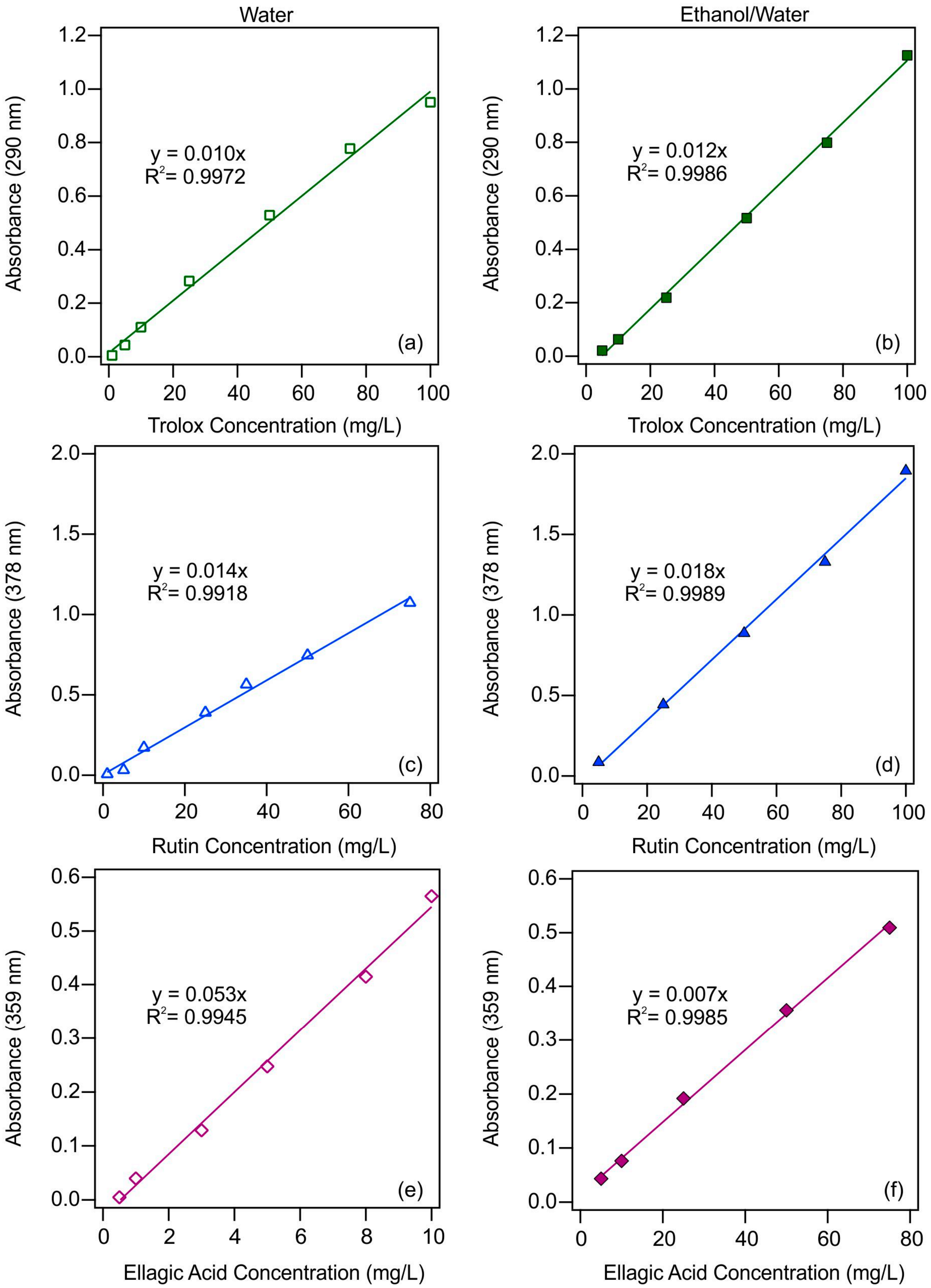

Figure S1. Calibration curves for antioxidants in water (left column) and in ethanol/water (right column) obtained at the wavelength corresponding to the absorption maxima determined for each antioxidant.

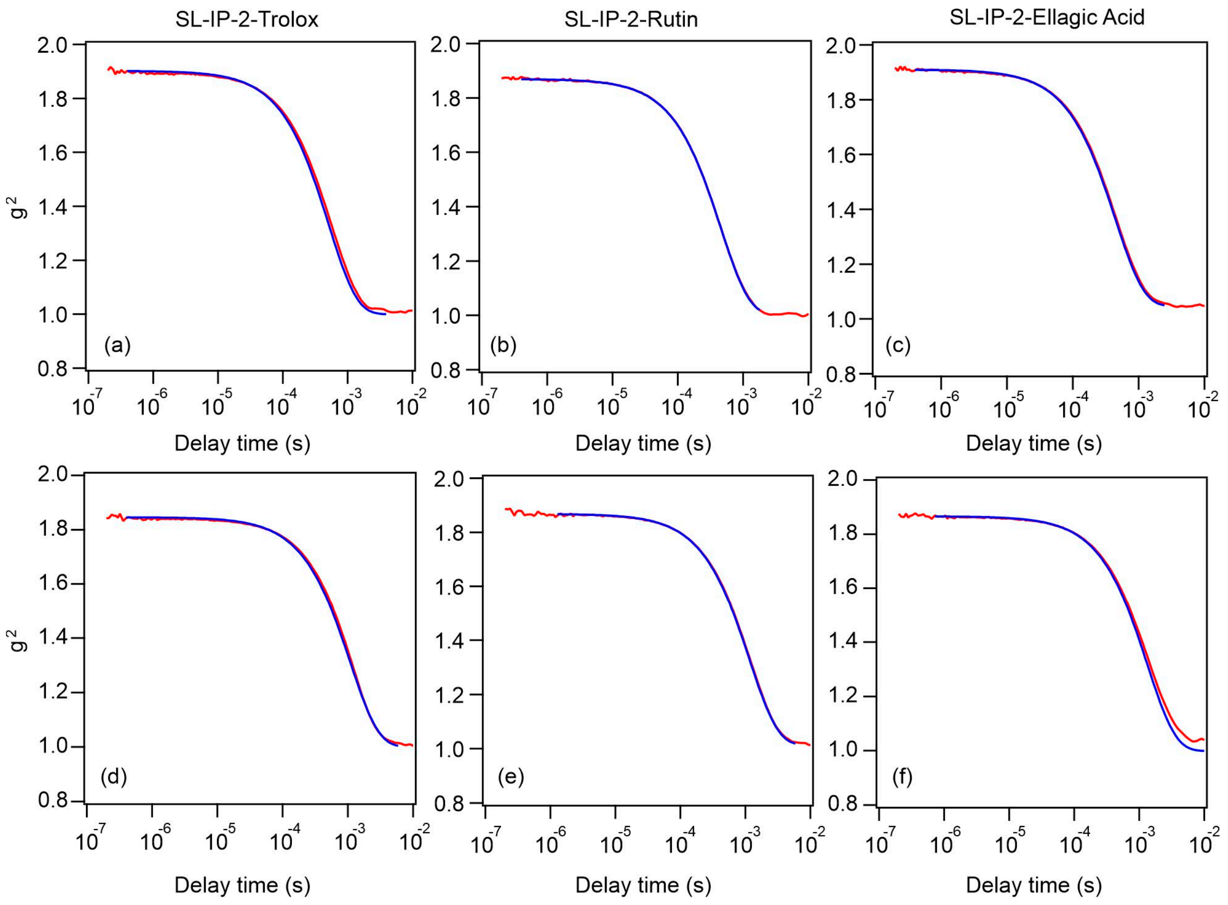

Figure S2. Intensity correlation function (g^2) versus the delay time for SL-IP-2-Trolox, SL-IP-2-Rutin, and SL-IP-2-Ellagic acid systems in water (a-c), and in ethanol/water (d-f). The antioxidant dose in all systems was maintained at 100 mg/g. The red solid line refers to the correlation function, and blue solid line represents the Cumulant fit.

Table S1. Characteristic size and charge data of the bare SL particles and SL-IP-2 in water.

Compound	R_h[a] (nm)	EM[a] (10^{-8} m^2/Vs)	PDI[a] (%)	IP-2 dose[b] (mg/g)
SL	137	-3.96	7.3	-
SL-IP-2	143	1.87	10.2	300

[a]Hydrodynamic radius (R_h), electrophoretic mobility (EM), and polydispersity index (PDI) were measured by light scattering at 1 mM ionic strength. The average errors of the measurements are ± 6 nm and ± 3.2% for the R_h and PDI, respectively, while for the electrophoretic mobilities the average error is about 10%. [b]IP-2 dose refers to the polymer concentration in terms of mg of IP-2 per 1 g of SL.

Table S2. The hydrodynamic radius (R_h), polydispersity index (PDI), and electrophoretic mobility (EM) data of SL-IP-2 with 100 mg/g dose of antioxidants. Effective concentrations and minimum DPPH% values are also presented.

System	SL-IP-2-Trolox		SL-IP-2-Rutin		SL-IP-2-Ellagic Acid	
Antioxidant structure	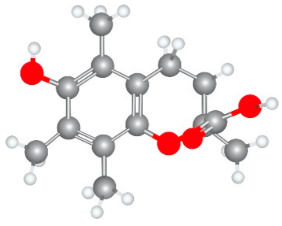		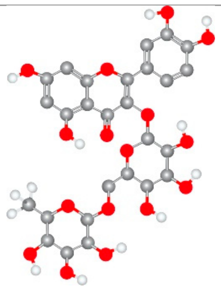		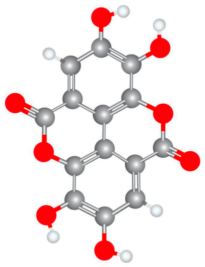	
solvent	H_2O	EtOH/H_2O	H_2O	EtOH/H_2O	H_2O	EtOH/H_2O
[a]*R_h* *(nm)*	147	431	142	426	142	476
[a]*PDI (%)*	6.88	12.11	7.64	14.70	13.87	18.78
[b]*EM* *(10^{-8} $m^2V^{-1}\cdot s^{-1}$)*	0.42	1.53	0.48	1.51	0.59	1.43
[c]*EC_{50}* *(mg/L)*	-	3.0	-	21.2	-	0.9
minimum *DPPH%*	54.8	0.3	60.1	47.3	89.6	5.4

[a]Hydrodynamic radius (R_h) and polydispersity index (PDI) values were determined by DLS. The measurement errors are ± 7 nm, and ± 2.50% for R_h and PDI, respectively. [b]Electrophoretic mobility (EM) was measured by light scattering in 1 mM KCl concentration with an average error of 10%. [c]The effective concentration (EC_{50}) values were determined in the DPPH assay with an average error of 5%.
